# Quetiapine as add-on treatment for bipolar I disorder: efficacy in preventing relapse of depressive episodes

**DOI:** 10.1186/1745-0179-3-17

**Published:** 2007-09-24

**Authors:** Maria Carolina Hardoy, Alessandra Garofalo, Gisa Mellino, Francesco Tuligi, Mariangela Cadeddu, Mauro Giovanni Carta

**Affiliations:** 1 Center for Epidemiology and Clinical Practice in Mental Health ASL 7 Iglesias, Italy and Department of Public Health, University of Cagliari, Italy; 2 AUSL, Lanusei, Italy

## Abstract

**Objective:**

To assess the long-term response to add-on quetiapine therapy in patients with bipolar I disorder who were not adequately responding to standard medications.

**Methods:**

Outpatients with bipolar I disorder (DSM-IV-TR) responding inadequately to standard treatment were observed before and after the addition of quetiapine. Symptom severity was evaluated using the Clinical Global Impressions scale for Bipolar Disorder (CGI-BP) each month. Relapses included hospitalization, treatment in a day hospital or clinic, scores ≥ 1 point higher than previous CGI-BP scores and/or upward titration of quetiapine or other medications.

**Results:**

Sixty-one patients (age range of 18–68 years) were observed prospectively for an average of 7.5 months (range 3–18 months) prior to addition of quetiapine and subsequently followed for an average of 15.7 months (range 6–42 months). The final mean quetiapine dose was 537.1 ± 91.7 mg/d. Prior to quetiapine addition, an annual relapse rate of 2.09 episodes was recorded, relating to 0.94 depressive and 1.15 manic or mixed episodes. Following quetiapine addition, annual relapse rates were reduced to 0.61 episodes, representing 0.14 depressive and 0.46 manic or mixed episodes. Compared with the period of add-on quetiapine treatment, the relative risk of relapse *prior *to quetiapine therapy was 3.4 for all episodes (χ^2 ^= 24.8, *P *< 0.001), 6.7 for depressive episodes (χ^2 ^= 24.7, *P *< 0.001), and 2.5 for manic or mixed episodes (χ^2 ^= 9.0, *P *< 0.05).

**Conclusion:**

This naturalistic follow-up study provides preliminary evidence for the efficacy of long-term add-on quetiapine treatment in the prevention of relapses of manic or mixed and depressive episodes of bipolar I disorder, and particularly in the prevention of depressive episodes.

## Background

Bipolar I disorder is a severe and chronic illness characterized by episodes of mania and depression [1]. Two major challenges of treating bipolar I disorder are the high percentage of patients who do not respond to therapy and the high percentage of patients who relapse after initially responding. To address these challenges, patients are often given long-term treatment with combinations of drugs from different classes [2]. Quetiapine is an atypical antipsychotic with a superior tolerability profile to conventional antipsychotics. Large, placebo-controlled studies have shown the efficacy of quetiapine for treating both acute manic episodes (as monotherapy and combination therapy) and acute depressive episodes (as monotherapy) associated with bipolar disorder [3-6].

### Objective

To assess the long-term response to add-on quetiapine therapy in patients with bipolar I disorder who were not adequately responding to standard medications.

## Methods

### Study design

An open-label study of patients with bipolar I disorder inadequately responsive to ongoing medications who were prospectively observed for 3–18 months before receiving add-on quetiapine treatment for 6–42 months.

### Study population

Adult outpatients with bipolar I disorder (based on DSM-IV-TR) [7] who had responded inadequately to prior standard treatment. Inadequate response to prior treatment was defined as a Clinical Global Impressions scale for Bipolar Disorder (CGI-BP) [8] score ≥ 3 with no improvement in score after 3 months of therapy. Patients who were pregnant or breastfeeding, or had a recent history of alcohol or drug abuse, were excluded. Written consent for the study was obtained after giving patients a complete description of the study.

### Study medication

Quetiapine was added to ongoing medication at an initial dose of 25 mg/d for the first 2 days, increased to 50 mg/d for the next 2 days, and then increased by 50 mg increments every 2 days until a clinical response was observed (up to a maximum dose of 800 mg/d). This dose was then maintained throughout the remainder of the study.

### Assessments

Prospective evaluations were made at least once every 2 months and no fewer than 8 times per year. Clinical response was evaluated using the CGI-BP scale [8]. The relative risk of relapse, defined as the number of relapse events per patient-year of treatment, was determined for the period before initiating quetiapine. Relapse events included hospitalization, treatment in a day hospital or clinic, or an increase of ≥ 1 in CGI-BP [8] score accompanied by a change in therapy.

### Statistical methods

Mean CGI-BP scores were compared by one-way analysis of variance (ANOVA) for repeated measures. Confidence intervals for comparing the relative risks of relapse were calculated using the simplified method of Miettinen [9].

## Results

### Patient and treatment characteristics

Of the 61 patients, 41% were male (mean age 41.4 ± 8.2 years) and 59% were female (mean age 47.2 ± 16.9 years). Patients were prospectively observed for 3–18 months (average 7.5 months) before quetiapine therapy was added. Patients' ongoing medications are listed in Table 1. Add-on quetiapine therapy was maintained for 6–42 months (average 15.7 months) until study termination. Fourteen patients received quetiapine add-on therapy for ≥ 24 months. The final mean quetiapine dose was 537.1 ± 91.7 mg/day. Four patients discontinued the study: 1 due to adverse effects (hypotension and drowsiness) and 3 due to non-adherence after the first evaluation at 6 months.

**Table 1:** Ongoing medications*

Medication	Number of patients
Carbamazepine	8
Chlorpromazine	5
Oxcarbazepine	2
Gabapentin	3
Haloperidol	8
Lithium	25
Olanzapine	5
Risperidone	4
Sodium valproate	14

*Some patients were treated with more than 1 of the listed medications.

### Efficacy

#### Risk of relapse

**Figure 1:**
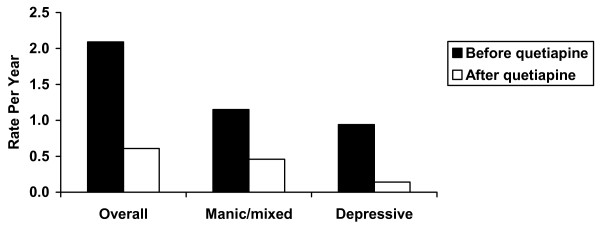
Annual relapse rates before and after add-on quetiapine treatment.

The overall relapse rate decreased following the addition of quetiapine (Table 2, Figure 1). When analyzed by episode type, the relapse rates of depressive and manic/mixed episodes also decreased after adding quetiapine compared with the period before adding quetiapine (Table 2, Figure 1). Relative risks of relapse for all episodes, manic episodes, and depressive episodes prior to quetiapine treatment are shown in Table 2.

**Table 2:** Relapse rates and relative risks of relapse before and after the addition of quetiapine to ongoing therapy

	Before add-on quetiapine	After add-on quetiapine
Observation period (in months)		
Average	7.5	15.7
Range	3–18	6–42
Relapse rate (episodes/year)		
Any episode	2.09	0.61
Manic/mixed episodes	1.15	0.46
Depressive episodes	0.94	0.14
Relative risk of relapse before add-on quetiapine		
Any episode	3.4 (χ^2 ^= 24.8, 95% CI 2.1–5.5; P < 0.001)	
Manic/mixed episodes	2.5 (χ^2 ^= 9.0, 95% CI 1.4–4.5; P < 0.05)	
Depressive episodes	6.7 (χ^2 ^= 24.7, 95% CI 3.7–14.0; P < 0.001)	

#### Symptom improvement

Mean change in CGI-BP score showed a significant improvement in symptoms from baseline at 6, 12, 18, and 24 months (*P *< 0.001; Table 3).

**Table 3:** Mean CGI-BP scores before and after addition of quetiapine

		CGI-BP Score (Mean ± SD)			
				
Observation period	N	Baseline*	During observation	F-ratio	***P*-value**^†^
3 months before quetiapine	61	4.5 ± 1.1	4.3 ± 1.4		
6 months after quetiapine	61	4.5 ± 1.1	3.4 ± 1.1	30.5	<0.001
12 months after quetiapine	47	4.7 ± 1.0	3.3 ± 1.3	34.3	<0.001
18 months after quetiapine	24	4.8 ± 0.9	3.6 ± 1.0	19.1	<0.001
24 months after quetiapine	14	4.4 ± 0.9	3.0 ± 0.9	16.9	<0.001

*Represents the start of quetiapine treatment except for the observation period at 3 months before the start of quetiapine treatment

^†^Observation period versus baseline

#### Tolerability

Side effects during quetiapine combination therapy (Table 4) were generally mild or moderate. Mild extrapyramidal symptoms (EPS) were reported by 4 patients (6.5%), all of whom were taking lithium or divalproex. No tardive dyskinesia was reported.

**Table 4:** Side effects during add-on quetiapine treatment

Adverse event	Patients, n (%)
Weight gain (> 7% of baseline body weight)	21 (34.4%)

Sedation	15 (24.6)
Asthenia	7 (11.4)
Insomnia	7 (11.4)
Dry mouth	5 (8.2)
Transient drowsiness	4 (6.5)
Headache	4 (6.5)
Constipation	3 (4.9)
EPS	4 (6.5)

## Conclusion

In patients with bipolar I disorder who had shown inadequate responses to prior standard therapy, relapse rates and symptoms were significantly improved with 6 months of add-on quetiapine therapy. These improvements were maintained in 14 patients treated for 24 months. Add-on quetiapine therapy was well tolerated, with no incidences of tardive dyskinesia reported following addition of quetiapine and only 4 patients reporting mild EPS. This naturalistic follow-up study demonstrates the efficacy of quetiapine in the prevention of relapses of manic and depressive episodes of bipolar I disorder in the long term, and particularly in the prevention of depressive episodes, which is consistent with our earlier findings [10] and with other follow-up studies concerning bipolar depression [11], bipolar depression and rapid cycling disease course [12], rapid cycling bipolar disorders [13]. These results warrant confirmation in large, randomized, placebo-controlled studies.
